# Gellan Gum-Based Hydrogel for the Transdermal Delivery of Nebivolol: Optimization and Evaluation

**DOI:** 10.3390/polym11101699

**Published:** 2019-10-16

**Authors:** Anroop B. Nair, Jigar Shah, Bader M. Aljaeid, Bandar E. Al-Dhubiab, Shery Jacob

**Affiliations:** 1 Department of Pharmaceutical Sciences, College of Clinical Pharmacy, King Faisal University, Al-Ahsa 31982, Saudi Arabia; baldhubiab@kfu.edu.sa; 2 Department of Pharmaceutics, Institute of Pharmacy, Nirma University, Ahmedabad 382481, Gujarat, India; jigsh12@gmail.com; 3 Department of Pharmaceutics, Faculty of Pharmacy, King Abdulaziz University, Jeddah 21589, Saudi Arabia; baljeaid6684@gmail.com; 4 Department of Pharmaceutical Sciences, College of Pharmacy, Gulf Medical University, Ajman 4184, UAE; sheryjacob6876@gmail.com

**Keywords:** nebivolol, gellan gum, factorial design, gel, pharmacokinetics

## Abstract

Poor solubility and appreciable first-pass metabolism have limited the oral bioavailability of nebivolol. The objective of the current investigation was to design, formulate, and optimize a hydrogel-based transdermal system for nebivolol using factorial design and compare its pharmacokinetics with oral suspension. Hydrogel formulations (F1–F8) were prepared by varying the amounts of gellan gum, carbopol, and polyethylene glycol. A 2^3^ full factorial design was used to assess the effect of independent variables such as gellan gum, carbopol, and polyethylene glycol 400 on dependent variables like viscosity, *in vitro* release, and *ex vivo* permeation after 2 h at two levels. Optimized gel (F7), containing nebivolol hydrochloride (75 mg), gellan gum (300 mg), carbopol (150 mg), polyethylene glycol 400 (20 µL), tween 80 (1 mL), ethanol (10 mL), and water (up to 30 mL) was selected and evaluated in albino rats. The physicochemical properties of F7 (pH: 7.1 ± 0.15, viscosity: 8943 ± 116 centipoise, drug content: 98.81% ± 2.16%) seem ideal for transdermal application. It was noticed that the concentration of carbopol has a more significant role than gellan gum in gel viscosity. A biphasic release pattern was exhibited by gels, and the release rate was mainly influenced by the concentration of gellan gum. Greater transdermal flux (30.86 ± 4.08 µg/cm^2^/h) was observed in F7 as compared with other prepared gels. Noticeable enhancement in *AUC*_0-α_ value (986.52 ± 382.63 ng.h/mL; *p* < 0.01) of transdermal therapy (~2-fold higher compared with oral administration) established the potential of F7 to improve the rate and extent of nebivolol delivery. The overall results demonstrated here signify that F7 could be a feasible alternative to oral therapy of nebivolol.

## 1. Introduction

Hypertension remains among the most serious health risks for millions of human populations. The World Health Organization’s (WHO) statistical report indicates that more than one billion people have been diagnosed with hypertension and estimated the increase in the global incidence to be ~60% more by 2025 [1]. Regrettably, high blood pressure is the primary contributing cause of global mortality and kills millions of people yearly [2]. It is a well-documented fact that hypertension is identified with enhanced risk of cardiovascular diseases such as heart failure, myocardial infarction, and stroke. Thus, lowering of blood pressure to normal values is the most important way of reducing cardiovascular risk. Various classes of drugs are available for the management of acute and chronic hypertension, which reduces the elevated blood pressure to normal levels by different mechanisms. Despite the availability and use of various antihypertensive agents, globally ~10 million people still have hypertension, and the effective management and treatment of hypertension still remains elusive.

Among various antihypertensive agents, beta blockers are well established and preferred in people with heart failure as they have shown to reduce the mortality [3]. Furthermore, these drugs are beneficial in patients with past myocardial infarction and in the management of angina pectoris, primarily due to their potential to reduce cardiac output [4]. The major limitations of traditional β blockers is their failure to be cardioselective and exclusively inhibit β_1_ receptors of the myocardium [5]. On the other hand, the new-generation drugs like nebivolol are promising, as they are very specific to β_1_ adrenoceptors, show extra vasodilation, and are most effective in reducing raised blood pressure. Nebivolol is a competitive and highly cardioselective β_1_ receptor blocker which induces endothelium-dependent vasodilation through nitric oxide release [6]. This drug possesses distinct pharmacodynamic characteristics and provides additional benefits, including better response and less adverse effects [7]. Moreover, it is a long-acting antihypertensive agent preferred for treating chronic heart failure in high-risk patients [8]. Given the therapeutic significance, nebivolol was approved by United States Food and Drug Administration in 2007 and since then, it is intensively used for the management of hypertension [9]. However, the efficiency of nebivolol in oral therapy was reported to be extremely low (~12% oral bioavailability) [10]. This low bioavailability is primarily because of its poor solubility in gastric fluid (0.176 mM), which in turn reduces the dissolution rate, in addition to the extensive first-pass hepatic metabolism it undergoes. The biopharmaceutical classification system (BCS) has categorized nebivolol under class II (low solubility and high permeability), suggesting that the non-oral route can be a feasible alternative.

Transdermal delivery, a noninvasive approach, is considered as an alternate route to oral and parenteral administration of drugs, wherein the drug is applied on the skin surface for the systemic therapy. This drug delivery system could be a possible substitute to oral therapy for those drugs which have specific issues including stability, absorption, dissolution, and first-pass metabolism [11]. Numerous pharmaceutically active compounds are successfully delivered through the skin, in a clinically relevant concentration, using different transdermal systems [12]. The selection of the transdermal system is based on both the drug’s physicochemical properties and its therapeutic application [13]. On the other hand, traditional transdermal systems are suitable for small, lipophilic and low-dose drugs while fail to deliver macromolecules [14]. Consequently, both physical and chemical approaches have been proposed and developed to improve the transdermal flux of drug molecules using electricity, ultrasound, heat, microneedle, chemical enhancers, as well as carrier systems like micro/nanoemulsions, elastic ultradeformable vesicles including liposomes, transfersomes, invasomes, ethosomes, and niosomes [15]. In the case of nebivolol, the physicochemical characteristics such as partition coefficient (log P 3.2) favor its percutaneous absorption. Furthermore, the low dose (<10 mg) as well as half-life (~10 h) suits this drug as a candidate for transdermal delivery. Being a BCS class II drug, a suitable drug delivery system which can load an adequate amount of nebivolol and release it on the skin surface is good for its transdermal therapy. In this context, gel formulations like hydrogels could be more appropriate for nebivolol transdermal therapy because of its low dose. Literature signifies that gel formulations like hydrogels, organogels, and emulgels have consistently been studied for delivering pharmaceutical actives through the skin [16,17,18]. Hydrogel, a versatile drug delivery system, has emerged as an attractive delivery mode for transdermal therapy of drugs because of the physicochemical and biological characteristics it possesses, which includes controlled drug release, good stability, higher percutaneous absorption, and nontoxic nature. In addition, hydrogels can offer the advantages of flexibility in dosing, ease of application, reduce skin irritation, promote skin hydration, improve drug diffusion, and improve patient compliance. Among various polymers studied, gellan gum, an anionic polysaccharide, has gained much attention in recent years owing to its excellent gelling and tunable mechanical properties [19]. Various transdermal formulations including semisolid gels, hydrogel films, and nanogels were successfully fabricated using gellan gum [20,21]. Low-acyl gellan gum is a natural anionic polysaccharide consisting of glucuronic acid, rhamnose, and glucose and is an ideal gelling agent [22]. It is a high-molecular-weight, water-soluble polymer, which easily disperses in aqueous media and provides good stability and high gel strength at low concentrations in the presence of gel-promoting cations [23]. On the other hand, carbopol 940 is a crosslinked polyacrylic acid polymer which forms clear gels that are ideal for transdermal applications [24]. This large-molecular-weight carbomer is also an excellent rheology modifier, resulting in greater residence time at the site of administration. Primary investigation revealed that the combination of gellan gum (gelling agent) and carbopol 940 (viscosity modifier) form a stable hydrogel with an adequate gelling property for transdermal delivery. Another formulation component (co-solvent) used is polyethylene glycol 400 (PEG 400). It is a clear, viscous, nonvolatile, nonirritating, low-molecular-weight polyether compound composed of polyethylene and water. It is widely used as a solvent and permeation enhancer in transdermal formulations [25]. Tween 80, a nonionic surfactant, is a solubilizing agent for a variety of substances and is included in the formulation to enhance the solubility of nebivolol. The objective of this investigation was to design, formulate, and optimize a hydrogel transdermal system using factorial design for the effective delivery of nebivolol. Optimization was performed by examining the effect of various formulation components (gellan gum, carbopol, and PEG 400) on viscosity, *in vitro* release, and *ex vivo* permeation. The optimized gel (F7) contained nebivolol HCl (75 mg), gellan gum (300 mg), carbopol (150 mg), PEG 400 (20 µL), tween 80 (1 mL), ethanol (10 mL), and water (up to 30 mL). The optimized hydrogel was assessed for the transdermal efficacy through *in vivo* studies and compared with oral suspension in albino rats.

## 2. Materials and Methods

### 2.1. Materials

Nebivolol hydrochloride was donated by Zydus Cadila, Ahmedabad, India. Low-acyl gellan gum (GELRITE; MW 2.5 × 10^5^ Da) was purchased from Duchefa Biochemie, Haarlem, Netherlands. PEG 400, acetonitrile, ethanol, and tween 80 were procured from Sigma Aldrich, St. Louis, MO, USA. Carbopol 940P was purchased from Yarrow Chem Product, Mumbai, India.

### 2.2. HPLC Determination of Nebivolol

Estimation of nebivolol in samples was carried out by a high-performance liquid chromatography (HPLC) system (Jasco LC–4000, Jasco Inc., Easton, MD, USA). The HPLC system used is made up of a Purospher C18 endcapped (150 × 4.6 mm, 5 µm) with a MD–4010 UV–Visible absorbance detector. Chromatographic separation of nebivolol as well as carvedilol (internal standard) was attained by a mobile phase comprising acetonitrile and 0.2% (*v*/*v*) triethylamine in water (80%:20% *v*/*v*). The temperature in the column was kept at 25 °C, and the flow rate of the mobile phase was adjusted to 1 mL/min. The injection was made with a sample size of 50 µL, and the chromatogram was recorded at wavelength of 280 nm. Linear regression analysis indicated good linearity in the nebivolol concentration of 1–80 µg/mL (r^2^ = 0.999). A separate calibration curve was developed to quantify nebivolol in the rat plasma. Plasma was analyzed by HPLC for nebivolol concentration, after the proteins were precipitated by adding equal volume of acetonitrile and 2-propanol. Then, the samples were centrifuged at 8000 rpm (15 min), and the supernatant was filtered using 0.22 μm filter (syringe filter, Merck, India) and injected (50 μl) into the HPLC. The concentration of nebivolol in the range of 10–1000 ng/mL, showed good linearity (r^2^ = 0.998). The limit of quantitation and limit of detection of the analytical method was found to be 20.28 ng/mL and 6.69 ng/mL, respectively. The coefficient of variation and accuracy ranged 6.40%–8.66% and −2.27 to −7.19, respectively.

### 2.3. Fourier-Transform Infrared (FTIR) Spectroscopy

A preformulation study was performed to find out the probable drug excipient interactions and stability issues of the drug in gel components using FTIR spectroscopy. Nebivolol and physical mixtures of gel components were prepared by grinding dried samples and kept for 30 days at 25 ± 0.2 °C/75% ± 5% relative humidity in a stability chamber [26]. Then, the samples were dried (60 °C for 30 min) to remove moisture and mixed with KBr (1:5), and discs were prepared by a hydraulic press. Scanning was carried out between 400 cm^−1^ and 4000 cm^−1^ with a minimum of 16 scans per sample, using a Jasco FTIR spectrometer (FP–6500, Tokyo, Japan). Spectra of nebivolol and the physical mixture of drug and excipient (1:1) were compared for changes in drug peaks.

### 2.4. Formulation of Design Batches Using 2^3^ Full Factorial

The main objective behind the selection of a full 2^3^ factorial experimental design was to perform a thorough investigation on the influence of the factors like concentrations of gellan gum (*X*_1_), carbopol (*X*_2_), and PEG 400 (*X*_3_) and their interactions, utilizing a statistical analytical tool (Design-Expert^®^ DX 11 Software, StatEase Design Expert, Statistical software, MN, USA) by considering one-way ANOVA at a statistical significant level of 0.05. In 2^3^ factorial design, concentrations of gellan gum, carbopol, and PEG 400 were taken as independent variables and viscosity, *in vitro* release (after 2 h), and *ex vivo* permeation (after 2 h) were considered as dependent variables.

The composition of various gels is mentioned in Table 1. Briefly, the required amounts of gellan gum, carbopol, and PEG 400 were dispersed in distilled water with continuous stirring on a magnetic stirrer (MS–4, Deepali United, Ahmedabad, India) at temperature maintained around 75 °C, until a uniform dispersion was formed. After the complete hydration of the polymers, drug (previously dissolved in ethanol and tween 80) was added with continuous stirring to obtain a transparent solution (Table 1). The mixture was cooled to room temperature with continuous stirring, using a magnetic stirrer. Calcium chloride, a divalent cation, (250 µL at 0.5 mM) was incorporated as a cross-linking agent to promote gelation of ion-sensitive low-acyl gellan gum. Triethanolamine (1 mL at 0.5%) was added to neutralize carbopol and stirred using homogenizer (Labindia, Mumbai, India) for 30 min to obtain the gel.

A statistical model employing interactive and first-order polynomial terms was utilized to check the response, as given by the following equation.

*Y* = *b*_0_ + *b*_1_*X*_1_ + *b*_2_*X*_2_ + *b*_3_*X*_3_ + *b*_4_*X*_1_*X*_2_ + *b*_5_*X*_1_*X*_3_ + *b*_6_*X*_2_*X*_3_

where *Y* is the dependent variable, while *b*_0_ is the intercept, *b*_1_, *b*_2_, *b*_3_, *b*_4_, *b*_5_, and *b*_6_ are regression coefficients; *X*_1_, *X*_2_ and *X*_3_ are main effects; *X*_1_*X*_2_, *X*_2_*X*_3_, and *X*_1_*X*_3_ are interactions between main effects.

### 2.5. Evaluation of Gels

The texture of the prepared gels was determined in terms of stickiness and grittiness by gently rubbing the gel between fingers. The appearance of gels was visually checked for transparency. The pH of prepared gels was recorded at room temperature using a digital pH meter (pHep, Hanna Instruments, Woonsocket, RI, USA). Viscosity of prepared gel formulations was measured with a viscometer (Brookfield LVDV-I Prime, Brookfield Engineering, Middleboro, MA, USA), using spindle number S96 at different rotation at 25 °C. The viscosity of prepared gels was carried out using new samples each time.

#### 2.5.1. Differential Scanning Calorimetry (DSC)

The thermal properties of pure drug and optimized gel (F7) were analyzed using DSC 60 (Shimadzu, Kyoto, Japan). Briefly, weighed drug or gel was taken in an aluminum pan, pressed against the bottom of the pan to ensure good contact between the sample and the bottom of the pan, and then crimp-sealed. An empty aluminum pan at the same experimental condition was used as reference during thermal analysis. The thermal scan of the prepared samples was performed between the temperature ranges of 25–300 °C at a heating rate of 5 °C/min in an inert nitrogen atmosphere.

#### 2.5.2. Drug Content

An accurately weighed quantity of gel (1 g) was taken in a polypropylene centrifuge tube, and mobile phase was gradually added. The solution was mixed in a mechanical shaker (EIE 405, EIE Instruments, Ahmedabad, India) for 1 h to get a homogenous solution, and then centrifuged at 4280× *g* for 10 min (R-83; Remi, Mumbai, India). Finally, the supernatant solution (1 mL) was passed through a 0.22 µm polyvinylidene syringe membrane filter (Millipore, Bedford, MA, USA). The amount of drug in the solution was estimated by HPLC after suitable dilution.

### 2.6. Drug Release

The *in vitro* nebivolol release from gels was carried out using Franz diffusion cell (Electro Lab, Mumbai, India), as described earlier on drug release from a topical gel formulation [27]. The active surface area for drug release was 1.13 cm^2^. A dialysis membrane with 2.4 nm pore size was soaked and fixed between donor and receptor compartment [28]. The receiver chamber (20 mL) contained phosphate-buffered saline (PBS; pH 7.4) plus tween 80 (10% *w/v*) as a surfactant to increase the solubility of nebivolol in the receiver media. This optimizes the release of nebivolol under sink conditions at a concentration of 50 µg/mL. The temperature of receptor media was set at 32 ± 0.5 °C and stirred constantly (50 rpm). Gel formulation (1 g) was applied on the surface of diffusion membrane and covered with Parafilm. Aliquot samples (5 mL) were withdrawn at different time periods (0.5, 1, 2, 3, and 4 h), and media was replaced with equal volume each time to maintain sink condition. The samples withdrawn were filtered through a 0.22 µm polyvinylidene syringe filter, and drug content was estimated using HPLC. The nebivolol release data were fitted into established mathematical release kinetics models and analyzed to predict the drug release pattern of formulated gels. The correlation coefficient (r^2^) as well release mechanisms were determined using following equations [29,30];

(a) Zero-order model *Q* = *Q*_0_ + *kt*

(b) First-order model *Q* = *Q*_0_ × e*^kt^*

(c) Higuchi model *Q* = *k* × *t*^0.5^

(d) Korsmeyer–Peppas model *Q* = *k* × *t^n^*

(e) Weibull model *Q* = 1 − exp[−(*t*)*^b^*^/*a*^]

where *Q* represents the quantity of drug released in time *t*, *Q*_0_ represents the value of *Q* at zero time, *k* represents the rate constant, *n* represents the diffusional exponent, *a* represents the time constant, and *b* represents the shape parameter. The correlation coefficient (*r*^2^) and the order of release pattern were calculated in each case.

### 2.7. Ex Vivo Permeation

The full thickness rat skin was obtained by surgically excising the fat using scalpel and scissors. The experimental setup in release studies was used here as well. The skin was fixed so that the stratum corneum side faces the donor compartment. Gel formulation (1 g) was applied on the surface of diffusion membrane and covered with Parafilm. The temperature of receptor media was set at 37 ± 0.5 °C and stirred constantly (50 rpm). Samples (5 mL) were withdrawn at different time periods (1, 2, 4, and 6 h), and media was replaced with equal volume each time. The amount of nebivolol in the receiver solution was estimated by HPLC. The steady-state flux, Jss (µg/cm^2^/h), was calculated from the slope of linear curve, plotted between nebivolol permeated through the skin area of 1 cm^2^ versus time [31].

### 2.8. In Vivo Studies

Male albino rats (150–250 g) placed in an animal house were fed ad libitum and kept under observation for 24 h. The ethical guidelines of the Institutional Animal Ethics Committee were strictly followed while performing experiments (IP/PCEU/FAC/23/2018/030). All rats were randomly classified into two groups, and each group consisted of six animals. Group 1 rats were anesthetized with phenobarbitone (30 mg/kg; intraperitoneally), and the hair in dorsal area was removed using an electric trimmer, and the administration area was cleaned using phosphate buffer saline. An open-ended circular holder (diameter 1 cm) with wings was fixed on the skin surface, optimized gel (F7) was applied uniformly (1 g in 1 cm^2^ area containing 2.5 mg of nebivolol) and was covered with Parafilm. For the second group, nebivolol suspension in phosphate buffer saline (1 mL) was administered perorally (1 mg/kg of nebivolol) using intragastric gavage [32]. The dose of nebivolol was calculated according to the standard human dose (10 mg), using an equation described in the literature [33]. At scheduled time periods (1, 2, 4, 6, 8, 12, and 24 h), blood samples were withdrawn (~200 µL) from retro orbital plexus and transferred into separate dry heparinized tubes. Dextrose was injected intraperitoneally (250 μl) after each blood sample was drawn.

### 2.9. Stability Studies

Gel F7 was stored in glass vials at 25 ± 0.2 °C/75% ± 5% relative humidity in a stability chamber for 3 months [34]. Samples were taken monthly and evaluated for physical appearance, pH, viscosity, assay, and release rate.

### 2.10. Skin Irritation

Skin irritation study was done for F7 as well as for standard skin irritant (2.5% sodium dodecyl sulfate). Formulation F7 was placed (100 mg) on both sides of the rat skin surface, and erythema and edema were scored for 24 h. The value of the test score was given based on Draize dermal irritation scoring criteria [35].

### 2.11. Data Analysis

Statistical analysis of data were carried out with GraphPad Prism (version 5, Graphpad software, San Diego, CA, USA), and values yielding *p* < 0.05 was treated as significant.

## 3. Result and Discussion

Initial studies were carried out to select suitable polymer (gellan gum or hydroxypropyl methylcellulose) and co solvent (PEG 400 or propylene glycol) to prepare hydrogel containing nebivolol. It was observed that the gels prepared using hydroxypropyl methylcellulose were translucent and had more air entrapment and high viscosity as compared with gellan gum-containing gels. On the other hand, the use of propylene glycol decreased the viscosity of gels as compared with PEG 400. Based on solubility studies and preliminary trial batches, gellan gum was selected as gelling agent, carbopol as viscosity modifier, PEG 400 as co-solvent, and tween 80 and ethanol as solubility enhancers.

### 3.1. FTIR Spectroscopy

**Figure 1:**
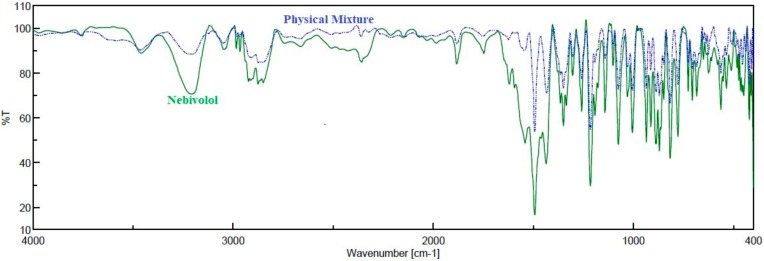
A representative FTIR spectra of nebivolol and physical mixture.

In preformulation studies, the possible interactions of drug with excipients and its influence on the stability of product is generally determined by the FTIR spectroscopy. The excipients in formulation components may interact chemically with drug moieties that can cause changes in molecular structure of the drug, which eventually influences the stability of the formulation [36]. Hence, FTIR spectra were recorded for nebivolol and the physical mixture of gel components and are depicted in the Figure 1. The FTIR spectrum of nebivolol depicts characteristic peaks at 3209.93 cm^−1^ (O–H stretching), 2873.42 cm^−1^ (C–H stretching), 1491.67 cm^−1^ (C=C stretching), 1349.93 cm^−1^ (C–N stretching), and 1141.67 cm^−1^ (C–O stretching). The spectra observed for the physical mixture indicate that all prominent characteristic peaks of the drug were present and there is no appreciable shift in band peak positions (Figure 1). These observations confirm the absence of any chemical interactions between nebivolol and gel components according to the literature [26].

### 3.2. Physicochemical Properties of Gels

Physicomechanical properties observed with F1–F8 are summarized in Table 2. Prepared gels were found to be transparent in appearance. Stickiness was observed in gels (F2 and F4), probably because of greater amount of gelling agent (gellan gum) and viscosity modifier (carbopol). A gritty nature was observed in gels (F1, F3, F6 and F8) where the total content of gellan gum and/or carbopol was less. Two gels (F5 and F7) were found to be nonsticky and nongritty. The pH of F1–F8 was measured to ensure the products do not cause any damage to the skin. The pH values (5.5–7.2) of F1–F8 in Table 2 seem to be ideal for transdermal application and are not likely to cause any irritations to the skin after application.

Estimation of drug content is a key quality control parameter of gel formulations as it confirms the availability of the drug and its uniform distribution in the product. The assay values in gels were in the range of 95–105% in F1–F8, and were not influenced by formulation ingredients in the current experimental settings.

### 3.3. DSC

**Figure 2:**
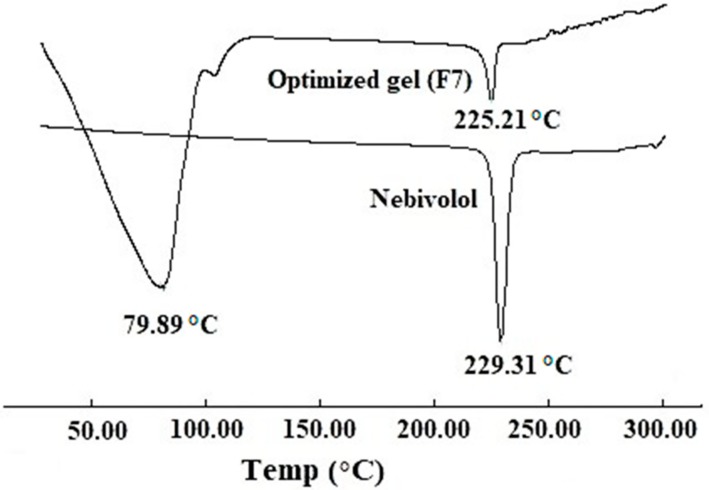
Representative differential scanning calorimetry patterns of nebivolol and optimized gel (F7).

DSC was carried out to assess the thermal and crystallization characteristics of nebivolol, and a representative thermogram corresponding to first heating run is shown in Figure 2. The thermogram of a native nebivolol shows a prominent and sharp endothermic peak at 229.31 °C, representing its melting point besides indicating its crystalline character. The thermogram of optimized gel (F7) shows the drug’s endothermic peak (225.21 °C). The amplitude of the DSC peaks is proportional to the weight fraction of each compound present in the sample, therefore it is evident that the peak of the drug is less intense in the gel (F7) since the analyzed sample is composed of drug, gellan, and other. The DSC data of the gel (F7) shows the presence of the solid drug in crystalline form, however this evidence might not be a limit, indeed it could be justified as a “reservoir” of solid drug in equilibrium with its solution. The additional endothermic peak (79.89 °C) observed in gel corresponds to the melting point of gellan gum.

### 3.4. The Effect of Formulation Variables on Viscosity

Consistency of gel plays an important role in transdermal application as the product should ensure adequate retention on the skin surface and release the drug in a controlled manner to provide effective delivery. A significant difference in viscosities of prepared gels were noticed with a change in carbopol amount (Table 2). From Table 2, it is evident that viscosity values were low in gels prepared with less carbopol content (150 mg). However, greater viscosity was noticed when the carbopol content was increased to 300 mg, probably due to the swelling behavior of carbopol [37], which is evidenced in gels F1–F4.

**Figure 3:**
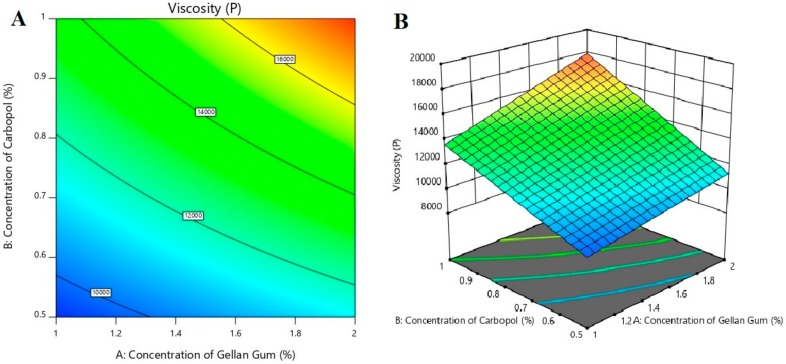
Effect of independent variables on viscosity using contour plot (**A**) and 3d response surface plot (**B**).

The three-dimensional (3d) response surface graphs and corresponding two-dimensional contour plots were generated by the Design-Expert^®^ DX 11 Software. The 3d graph is used for understanding interaction effects of the independent variables, whereas a two-dimensional plot is the expression of an individual response value [38]. The contour plot and 3d response surface plot (Figure 3) show the influence of independent variables on the viscosity of gels. From the graphs shown in Figure 3, it is confirmed that gellan gum and carbopol have a significant effect (*p* < 0.005) on the viscosity of gel formulations. Moreover, it is observed that the higher the gellan gum or carbopol concentration, the greater the viscosity of gels.

The fitted polynomial equation for viscosity is given below:

Viscosity = 13060.75 + 1538.50*X*_1_ + 2714.00*X*_2_ − 408.75*X*_3_ + 599.25*X*_1_*X*_2_ + 145.50*X*_1_*X*_3_ − 144.00*X*_2_*X*_3_.

where *X*_1_ is the concentration of gellan gum; *X*_2_ is the concentration of carbopol; *X*_3_ is the concentration of PEG 400. The main effects (*X*_1_, *X*_2,_ and *X*_3_) represent the mean outcome of varying one factor at a time, from low to high value. The interaction terms (*X*_1_*X*_2_, *X*_1_*X*_3_, and *X*_2_*X*_3_) indicate how the dependent variable was modified when two factors were simultaneously transformed. The positive sign of coefficient indicates that the variable has a favoring effect on viscosity, and the negative sign shows an opposite effect on viscosity of designed batches. Hence, gellan gum and carbopol have a positive effect (increase viscosity), while PEG 400 has a negative effect (decrease viscosity). The values in the above equation also suggest that the concentration of carbopol has a more significant effect than gellan gum on gel viscosity.

The simplification of the model was done by removing nonsignificant terms (*p* > 0.05) from the above equation [39], and the equation representing a reduced model was developed.

Viscosity = 13060.75 + 1538.50*X*_1_ + 2714.00*X*_2_ + 599.25*X*_1_*X*_2_.

### 3.5. Effect of Formulation Variables on Drug Release

**Figure 4:**
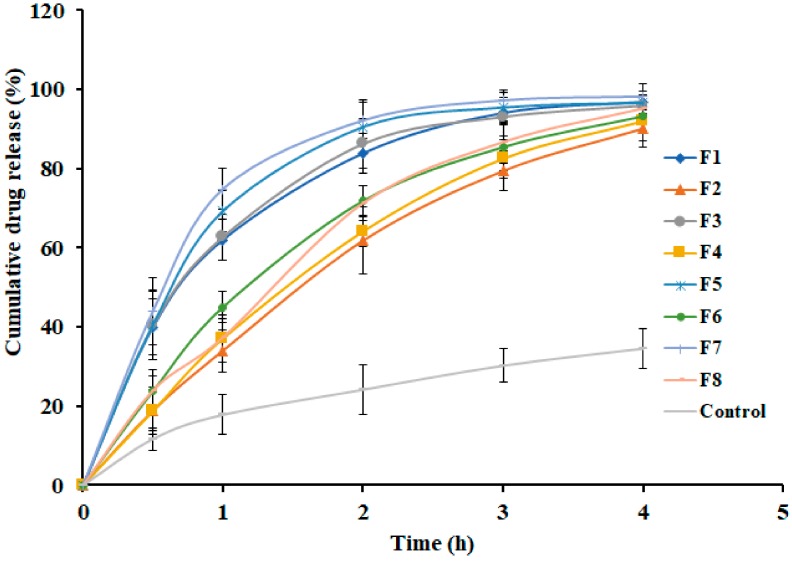
*In vitro* release profiles of nebivolol from prepared gels (F1–F8) at various time intervals. Data expressed as average ± SD (n = 6).

The release of drug molecules from prepared gel is necessary for their transport into and across the skin membrane to reach the systemic circulation, to exhibit therapeutic activity. However, the release of drugs from hydrogels are either by dissociation of actives inside the gel and then diffusion through the gel or by its liberation from the gel [40]. Figure 4 analyzes the cumulative percentage of nebivolol released from F1–F8 at specific time periods. It is evident from Figure 4 that the drug release from all the prepared gels (F1–F8) showed more than 90% in 4 h. Therefore, the period of the drug release testing was kept for 4 h. It can be seen from the Figure 4 that nebivolol release from prepared gels is biphasic, with a greater amount being released in the first hour itself. However, the amount of gellan gum influences the release rate of nebivolol. The drug release was relatively rapid (~60% in 1 h) from gels (F1, F3, F5, and F7) containing less concentration of gellan gum as compared with the gels (F2, F4, F6 and F8) that were prepared with a higher amount of gellan gum (Figure 4). This rapid release observed in the current study substantiates our choice of hydrogel for nebivolol transdermal therapy. On the other hand, variation in PEG content or carbopol did not significantly influence drug release in the current experimental conditions. However, the release profile of pure drug was found to be slow (Figure 4). The drug release data of the entire set were fit into various mathematical models to evaluate the kinetics and mechanism of release behavior. In the zero order, first, Higuchi, Korsmeyer–Peppas, and Weibull models used in release studies were curve-fitted to the observed experimental data. Various parameters like regression coefficient (r^2^), squares of residuals (SSR), and Fischer Ratio (FR) were used to predict the best fit and are presented in Table 3. The SSR and FR are two additional parameters used to reduce the error in predicting the release mechanism. A minimum value of these parameters indicates a good fit of the model to the data and minimum deviation from the actual data. Based on the above parameters, it is evidenced in Table 3 that the best fit for the release of nebivolol from the gels (F1–F8) is the Weibull release kinetics model. Therefore, the predominant mechanism for nebivolol release from these gels is diffusion, which is usually observed in transdermal gels.

**Figure 5:**
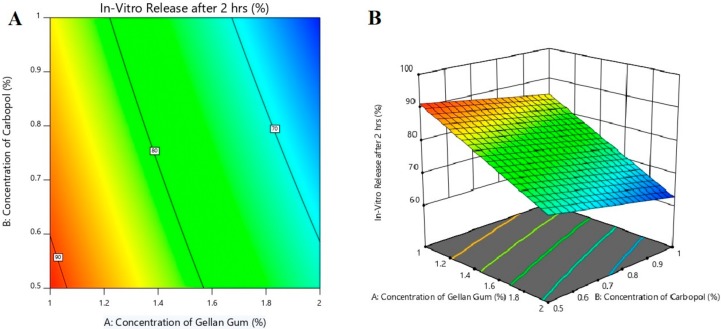
Effect of independent variables on *in vitro* release after 2 h using contour plot (**A**) and 3d surface plot (**B**).

Figure 5 shows the contour and 3d response surface plot of the effect of independent variables on *in vitro* drug release after the completion of 2 h. It also proves that the amount of gellan gum has a significant effect (*p* < 0.005) on release of nebivolol from gel formulations. As the concentration of gellan gum increases, the drug release decreases because of the viscous and swelling nature of gellan gum, which in turn could lead to lower diffusion of the drug through the swollen gel layer [41]. However, carbopol has very little and PEG 400 has no significant effect on the nebivolol release. The observed rapid drug release is certainly advantageous for transdermal delivery as this would facilitate the availability of a greater drug concentration on the gel–skin interface for permeation.

The polynomial equation for *in vitro* release after 2 h is shown below:

*In vitro* drug release after 2 h **=** 77.74 − 10.48*X*_1_ − 3.72*X*_2_ + 0.8625*X*_3_ − 0.5889*X*_1_*X*_2_ − 0.1428*X*_1_*X*_3_ + 0.3128*X*_2_*X*_3_.

The equation indicates that the concentration of gellan gum (*X*_1_) has a significant effect on *in vitro* drug release after 2 h as compared with carbopol (*X*_2_) and PEG 400 (*X*_3_). Gellan gum and carbopol have a negative effect (decrease drug release), while PEG 400 has a moderate positive effect (increase drug release), which is confirmed by the equation of main effects (*X*_1_, *X*_2,_ and *X*_3_) and interactive terms (*X*_1_*X*_2_, *X*_1_*X*_3_, and *X*_2_*X*_3_) value and sign.

The reduced model was developed by removing nonsignificant terms (*p* > 0.05), and the following equation was formed:

*In vitro* drug release after 2 h **=** 77.74 − 10.48*X*_1_ − 3.72*X*_2_.

### 3.6. Effect of Formulation Variables on Ex Vivo Permeation

**Figure 6:**
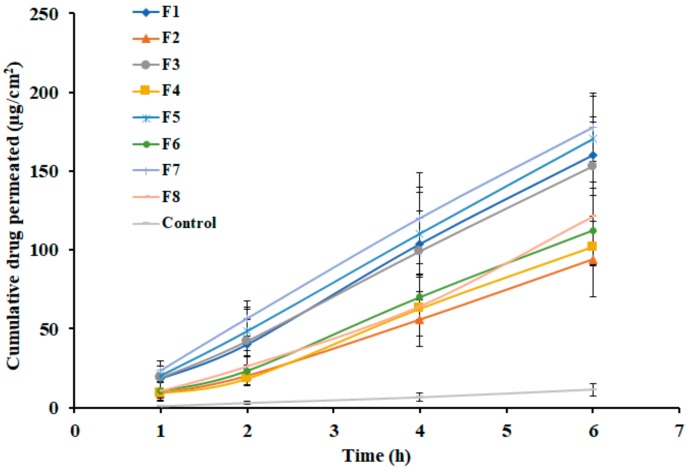
*Ex vivo* skin permeation profiles of nebivolol from prepared gels (F1–F8) at various time intervals. Data expressed as average ± SD (n = 6).

*Ex vivo* permeation study is generally conducted to predict the performance of drug transport into and through the skin membrane during *in vivo* use. In general, diffusion of the drug through biological barriers is influenced by various factors including drug physicochemical properties, physiological properties of the barrier, like thickness of the membrane and its composition, as well as the transport route accessible for the drug permeation [42,43]. The permeation potential of nebivolol was investigated using full-thickness rat skin. The amount of nebivolol diffused through the skin membrane from prepared gels (F1–F8) was determined and is depicted in Figure 6. It is evident from the Figure 6 that a certain amount of nebivolol (F1; 18.40 µg/cm^2^, F2; 9.04 µg/cm^2^, F3; 18.88 µg/cm^2^, F4; 9.16 µg/cm^2^, F5; 20.31 µg/cm^2^, F6; 10.43 µg/cm^2^, F7; 23.58 µg/cm^2^, and F8; 10.48 µg/cm^2^) has diffused into receiver fluid in one hour. The permeation parameters observed with different gels are outlined in Table 4. The lag time values observed in Table 4 with different gels signify quick permeation of nebivolol through the skin with a short lag time. Thereafter, the drug permeation consistently progresses with an increase in duration of time, in all the gel formulations. However, the flux values of nebivolol differ among gels (Table 4), and were relatively high from gels with low concentration of gellan gum (F1, F3, F5, and F7) as compared with gels prepared with higher gellan gum content (F2, F4, F6, and F8). This observation could be corroborated to the release data, where the amount of nebivolol liberation was higher when gellan gum concentration was low. On the other hand, the nebivolol permeation from control was slow and low, which again could be correlated to the low release profile observed in Figure 4.

**Figure 7:**
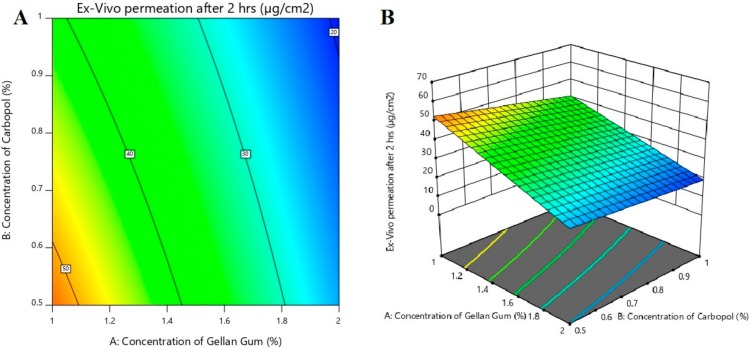
Effect of independent variables on *ex vivo* permeation (%) using contour plot (**A**) and 3d response surface plot (**B**).

The contour and 3d surface response plots shown in Figure 7 also indicated that as gellan gum concentration increases, drug permeation decreases. The responses, percentage drug release and amount of drug permeation were significantly higher only when gellan gum content was low. From the graphs shown in Figure 7, it is also confirmed that carbopol and PEG 400 have no significant effect on drug permeation from the formulations. The greater steady-state flux was noticed in gel F7 (~30.86 µg/cm^2^/h) and was low in gel F2 (~17.30 µg/cm^2^/h), among the tested gels. Based on the higher steady-state flux value, gel F7 was selected for *in vivo* studies.

The polynomial equation for *ex vivo* permeation after 2 h is shown below:

*Ex vivo* permeation after 2 h **=** 34.41 − 12.40*X*_1_ − 4.24*X*_2_ + 0.6870*X*_3_ + 1.49*X*_1_*X*_2_ − 1.86*X*_1_*X*_3_ − 0.6129*X*_2_*X*_3_.

The positive sign of coefficient indicates an increase in drug permeation, and the negative sign shows a decrease in drug permeation of designed batches.

The reduced model for *ex vivo* permeation after 2 h is;

*Ex vivo* permeation after 2 h **=** −12.40*X*_1_

### 3.7. In Vivo Studies

**Figure 8:**
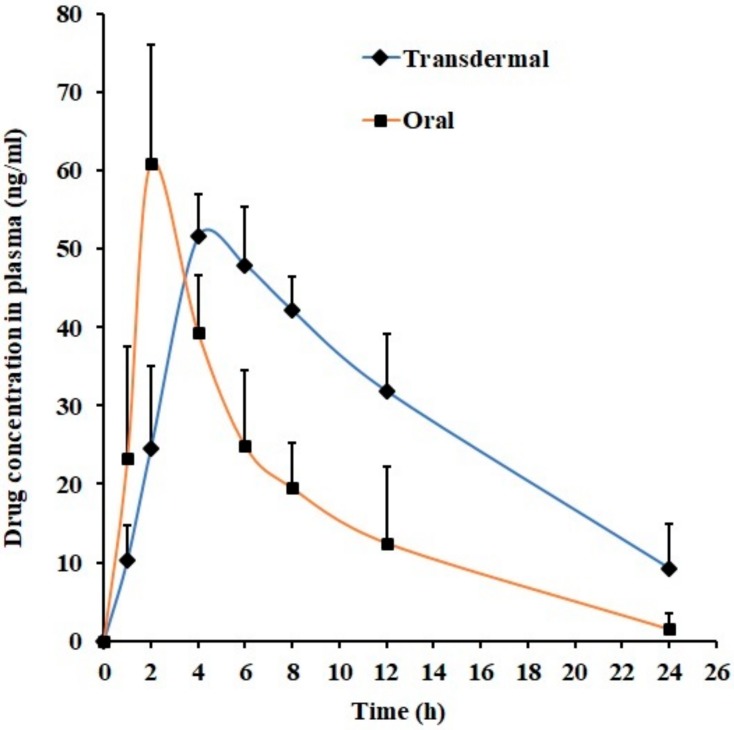
Plasma profiles of nebivolol at various time intervals in transdermal (gel F7) and oral administration in rats. Data expressed as average ± SD (n = 6).

In the final phase, *in vivo* studies were performed to compare drug pharmacokinetics parameters following its administration through the transdermal route (using optimized gel F7) and oral route in rats using non-compartment model. The pharmacokinetic parameters including maximum drug plasma concentration (*C*_max_) and time to achieve maximum plasma concentration (*T*_max_) were estimated by plotting drug plasma level versus time. The trapezoidal rule was applied to determine the area under the concentration–time curve from zero time to infinity (*AUC*_0-α)_ for individual dose administered. Figure 8 shows the mean plasma concentration versus time profile of nebivolol after transdermal and oral administration. The pharmacokinetic parameters mentioned in the Table 5 were determined from individual plasma versus time profiles. Figure 8 revealed that the mean plasma nebivolol profiles were relatively different when the route of administration was changed. Indeed, drug absorption was rapid and was detected in plasma at the initial time point (1 h) in both cases, though the nebivolol plasma level was different (transdermal; 10.33 ± 4.34 ng/mL, oral; 23.24 ± 14.34 ng/mL). In the case of transdermal administration of F7, the amount of nebivolol permeated through the skin and reaching blood was slow and low in the initial phase and was significantly different in 2 h (*p* < 0.001) as compared with oral delivery. In oral administration, absorption of nebivolol was rapid from gastrointestinal tract, with peak plasma concentration (*C*_max_; 60.95 ± 15.06 ng/mL) attained in 2 h (*T*_max_). However, the drug absorption was prolonged in transdermal administration, and the nebivolol level in plasma continued to rise up to 4 h (*T*_max_) with *C*_max_ (51.56 ± 5.41 ng/mL), which is not statistically significant when compared with oral administration. After the absorption phase, the drug plasma level declined slowly in both cases. It can be concluded from the Table 5 that the *AUC*_0-α_ value in transdermal application is ~2-fold higher (*p* < 0.01) when compared with oral suspension, demonstrating the potential utility of transdermal therapy of nebivolol. The data observed here signifies that optimized hydrogel could be a feasible alternative to oral therapy of nebivolol in the management and treatment of hypertension.

### 3.8. Stability and Skin Irritation

Data from stability studies shows no significant differences in physical appearance, pH, viscosity, and drug content of F7 during storage (3 months) when examined. Skin irritancy study was done to observe the possible cutaneous reactions of F7 and control, since safety on the skin is an essential criteria to be fulfilled by all transdermal products. Formulation F7 exhibited a Draize dermal scoring grade value of “0”, confirming it to be as nonirritant to the human skin [35]. No apparent erythema, edema, or inflammation was evident on the rat’s skin after application of F7 for one day when compared with standard irritant.

## 4. Conclusions

A systematic investigation was carried out to develop a transdermal system for effective delivery of nebivolol. Hydrogels (F1–F8) containing nebivolol were prepared by varying the amount of gellan gum, carbopol, and PEG 400. Optimization was carried out at different stages of the study by assessing the influence of formulation ingredients (gellan gum, carbopol, and PEG 400) on viscosity, *in vitro* release, and *ex vivo* permeation. *In vivo* data in rats suggest that transdermal therapy using optimized gel (F7) could be an effective alternative to oral therapy of nebivolol. The data demonstrated in the current investigation prove the feasibility of the optimized hydrogel (F7) to enhance the delivery of nebivolol across the skin.

## Figures and Tables

**Table 1:** Composition of nebivolol hydrogel formulations used in experiments.

Ingredients	Formulations							
	F1	F2	F3	F4	F5	F6	F7	F8
**Nebivolol HCl (mg)**	75	75	75	75	75	75	75	75
**Gellan gum (mg)**	300	600	300	600	300	600	300	600
**Carbopol (mg)**	300	300	300	300	150	150	150	150
**Polyethylene glycol 400 (µL)**	15	20	25	40	10	35	20	35
**Tween 80 (mL)**	1	1	1	1	1	1	1	1
**Ethanol (mL)**	10	10	10	10	10	10	10	10
**Water up to (mL)**	30	30	30	30	30	30	30	30

**Table 2:** Physicomechanical properties of prepared nebivolol hydrogel formulations.

Evaluation Parameters	Formulations							
	F1	F2	F3	F4	F5	F6	F7	F8
**pH**	5.69 ± 0.28	5.58 ± 0.35	5.78 ± 0.41	6.12 ± 0.25	6.9 ± 0.34	7.02 ± 0.29	7.1 ± 0.15	7.14 ± 0.22
**Viscosity (centi poise)**	14,281 ± 189	18,374 ± 224	12,993 ± 175	17,451 ± 152	9872 ± 80	11,221 ± 94	8943 ± 116	11,351 ± 127
Drug content (mg/g) *	2.41 ± 0.04	2.47 ± 0.06	2.49 ± 0.09	2.43 ± 0.05	2.45 ± 0.02	2.42 ± 0.04	2.47 ± 0.05	2.46 ± 0.06

* Milligrams of nebivolol per gram of gel.

**Table 3:** Model fitting for prepared nebivolol hydrogel formulations.

Formulations	Factors	Model Name				
		Zero order	First order	Higuchi	Korsmeyer – Peppas	Weibull Model
**F1**	**r^2^**	0.8080	0.9963	0.9703	0.9601	0.9989
	**SSR**	1353.74	39.66	209.26	130.11	0.88
	**FR**	338.44	9.91	52.32	32.53	0.22
**F2**	**r^2^**	0.9633	0.9909	0.9723	0.9915	0.9975
	**SSR**	231.14	135.18	174.73	76.12	6.13
	**FR**	57.79	33.79	43.68	19.03	1.53
**F3**	**r^2^**	0.7887	0.9855	0.9608	0.9469	0.9951
	**SSR**	1481.09	183.99	274.79	171.99	6.36
	**FR**	370.27	45.99	68.70	43.00	1.59
**F4**	**r^2^**	0.9559	0.9905	0.9733	0.9849	0.9992
	**SSR**	293.39	173.67	177.92	117.83	3.02
	**FR**	73.35	43.42	44.48	29.46	0.75
**F5**	**r^2^**	0.7481	0.9600	0.9374	0.8979	0.9778
	**SSR**	1868.63	413.18	464.57	313.73	30.92
	**FR**	467.16	103.29	116.14	78.43	7.73
**F6**	**r^2^**	0.9163	0.9984	0.9828	0.9721	0.9998
	**SSR**	565.37	32.12	116.34	158.36	0.7015
	**FR**	141.34	8.03	29.08	39.59	0.1754
**F7**	**r^2^**	0.7174	0.9753	0.9228	0.8733	0.9828
	**SSR**	2147.59	365.13	586.63	319.66	30.66
	**FR**	536.90	91.28	146.66	79.90	7.67
**F8**	**r^2^**	0.9401	0.9863	0.9751	0.9841	0.9886
	**SSR**	428.25	350.90	177.81	131.89	32.94
	**FR**	107.06	87.72	44.45	32.97	8.24

r^2^: Correlation coefficient; SSR: Sum of square of residuals; FR: Fischer ratio.

**Table 4:** *Ex vivo* permeation parameters observed in prepared nebivolol hydrogel formulations.

Formulations	Lag time (h)	Flux (µg/cm^2^/h)	Cumulative Amount Permeated (12 h) (µg/cm^2^)	Permeability Coefficient (cm/h ×10^−3^)
F1	0.46 ± 0.21	28.94 ± 2.66	160.15 ± 20.91	2.67 ± 0.39
F2	0.66 ± 0.29	17.30 ± 1.46	94.11 ± 24.02	1.57 ± 0.25
F3	0.36 ± 0.12	27.14 ± 2.52	153.14 ± 35.51	2.55 ± 0.32
F4	0.75 ± 0.22	19.25 ± 2.13	101.97 ± 29.75	1.70 ± 0.21
F5	0.35 ± 0.16	30.17 ± 3.56	170.37 ± 27.02	2.84 ± 0.45
F6	0.67 ± 0.30	20.95 ± 2.42	112.38 ± 22.09	1.87 ± 0.29
F7	0.19 ± 0.05	30.86 ± 4.08	177.76 ± 21.76	2.96 ± 0.22
F8	0.73 ± 0.25	22.05 ± 2.48	121.20 ± 31.51	2.02 ± 0.19

**Table 5:** Pharmacokinetic parameters of nebivolol in plasma following transdermal and oral administration in rats. *T*_max_, time of maximum concentration; *C*_max_ indicates maximum concentration; *AUC*_0-α_, area under the plasma concentration–time curve. Data expressed as average ± SD (n = 6), and *p < 0.05 were considered as significant.

Parameter	Transdermal Gel (F7)	Oral Suspension
*T*_max_ (h)	4	2
*C*_max_ (ng/mL)	51.56 ± 5.41	60.95 ± 15.06
*AUC*_0-α_ (ng.h/mL)	986.52 ± 382.63*	422.90 ± 192.64
